# Socioeconomic disparities in breast cancer incidence and survival among parous women: findings from a population-based cohort, 1964–2008

**DOI:** 10.1186/s12885-015-1931-4

**Published:** 2015-11-19

**Authors:** Mandy Goldberg, Ronit Calderon-Margalit, Ora Paltiel, Wiessam Abu Ahmad, Yechiel Friedlander, Susan Harlap, Orly Manor

**Affiliations:** 1 Department of Epidemiology, Columbia University Mailman School of Public Health, 722 W. 168th St., 7th floor, New York, NY 10032 USA; 2 Braun School of Public Health and Community Medicine, Hebrew University-Hadassah Medical Organization, Jerusalem, Israel; 3 Department of Hematology, Hebrew University-Hadassah Medical Organization, Jerusalem, Israel; 4 Department of Psychiatry, New York University School of Medicine, New York, NY USA; 5 Department of Obstetrics and Gynecology, New York University School of Medicine, New York, NY USA; 6 Department of Environmental Medicine, New York University School of Medicine, New York, NY USA

**Keywords:** Breast cancer, Incidence, Survival, Socioeconomic position, Education, Occupation, Disparities, Inequalities, Race/ethnicity, Population-based cohort

## Abstract

**Background:**

Socioeconomic position (SEP) has been associated with breast cancer incidence and survival. We examined the associations between two socioeconomic indicators and long-term breast cancer incidence and survival in a population-based cohort of parous women.

**Methods:**

Residents of Jerusalem who gave birth between 1964–1976 (*n =* 40,586) were linked to the Israel Cancer Registry and Israel Population Registry to determine breast cancer incidence and vital status through mid-2008. SEP was assessed by husband’s occupation and the woman’s education. We used log ranks tests to compare incidence and survival curves by SEP, and Cox proportional hazard models to adjust for demographic, reproductive and diagnostic factors and assess effect modification by ethnic origin.

**Results:**

In multivariable models, women of high SEP had a greater risk of breast cancer compared to women of low SEP (Occupation: HR 1.18, 95 % CI 1.03-1.35; Education: HR 1.39, 95 % CI 1.21-1.60) and women of low SEP had a greater risk of mortality after a breast cancer diagnosis (Occupation: HR 1.33, 95 % CI 1.04-1.70; Education: HR 1.37, 95 % CI 1.06-1.76). The association between education and survival was modified by ethnic origin, with a gradient effect observed only among women of European origin. Women of Asian, North African and Israeli origin showed no such trend.

**Conclusions:**

SEP was associated with long-term breast cancer incidence and survival among Israeli Jews. Education had a stronger effect on breast cancer outcomes than occupation, suggesting that a behavioral mechanism may underlie disparities. More research is needed to explain the difference in the effect of education on survival among European women compared to women of other ethnicities.

## Background

Social gradients in breast cancer have been observed within developed and developing nations [1–6]. Women of high socioeconomic position (SEP) have been found in most studies to have a higher risk of developing breast cancer [7–11] and higher rates of breast cancer mortality than women of low SEP, though recent studies show a diminishing social gradient in mortality in specific populations [12, 13], which may be due to narrowing differences in reproductive patterns by SEP [13] or increasing disparities in breast cancer survival by SEP [14]. Women of low SEP have been shown to experience poorer survival after diagnosis than women of high SEP [15–17].

In some studies, the association between SEP and breast cancer incidence was fully explained by factors such as age at first birth, parity, body mass index (BMI), oral contraceptive (OC) use and hormone replacement therapy use [8, 9, 11]. Others detected an excess risk of breast cancer among women of high SEP after controlling for these factors [2, 11]. Some studies found that the higher risk of mortality among women of low SEP is mediated by differences in diagnostic factors and tumor characteristics [18–20], while others found that the effect of SEP on survival is independent of these characteristics [16, 21]. The literature varies on the role that race/ethnicity plays in the associations between SEP and breast cancer outcomes [22, 23]. Some suggest socioeconomic inequalities in breast cancer are partially explained by racial/ethnic differences [24, 25], but the interplay between the three has not been extensively studied.

The inconsistencies in the literature in regards to the magnitude of the associations between socioeconomic indicators and breast cancer outcomes and the extent that these associations are explained by known risk factors may be due to differences in SEP measures. While indicators such as education, income and occupation are often used interchangeably as a proxy for SEP, they tend to be only moderately correlated and may capture different aspects of SEP, a multidimensional construct that includes resource- and status-based measures [26–28]. Studies that analyzed multiple socioeconomic indicators found that the associations with breast cancer outcomes differ depending on the indicator used [3, 10, 11, 15]. A number of studies have been conducted using area-level measures of SEP [20, 29–31], such as census tract rates, which are less precise than individual measures and may bias associations [27]. Few have examined the association between individual-level SEP and breast cancer incidence and survival within the same cohort, an approach that can determine if the reversed social gradient in incidence fully changes direction to a positive gradient in survival [2, 3]. Only one compared the effect of multiple socioeconomic indicators on incidence and survival [3].

We aimed to determine the association between SEP measured by occupation and education and long-term breast cancer incidence and survival in a population-based cohort, rich in demographic and reproductive data, and drawn from a population with high rates of breast cancer incidence and mortality. We also assessed whether ethnic origin modified the associations between SEP and breast cancer outcomes within this population.

## Methods

### Participants

The Jerusalem Perinatal Study prospectively followed a population-based cohort of 92,408 infants born to residents of Jerusalem between January 1964 and December 1976 and their parents. Detailed information on the cohort has been previously described [32–34]. The present investigation focused on mothers to Jewish infants (97.7 %). The identities of 40,586 women (96.9 %) from the original cohort were traced through the Israel Population Registry. They form the basis for this analysis.

### Data sources

Demographic and socioeconomic data were abstracted from birth notifications. Women were linked by their ID numbers to the population registry to verify demographic characteristics and assess vital status [34], and to the Israel Cancer Registry to assess breast cancer diagnoses. Linkage to the cancer registry occurred in 2010, when the registry was fully updated through July 2008. The registry was established in 1961 [35], and reporting of all cancer diagnoses, except non-melanoma skin cancer, has been mandatory since 1981 [36]. It is considered to be 94.2 % complete for breast cancer [37].

### Study variables

The main outcome variables were breast cancer diagnosis and mortality after diagnosis. Breast cancer diagnosis was defined by ICD-Oncology 3 codes 50.0-50.9. Entry to the cohort was defined as the date, within 1964 to 1976, that the woman first gave birth in Jerusalem, regardless of parity. Time to breast cancer diagnosis was defined as the time, in years, between cohort entry and the date of first breast cancer diagnosis or censored due to death or end of follow-up (July 31, 2008). For women diagnosed with breast cancer, breast cancer survival was defined as the time, in years, from the date of diagnosis to the date of death from any cause or censored due to end of follow-up (July 31, 2008).

SEP was assessed according to the occupation of the infant’s father and the woman’s educational attainment as recorded on the birth certificate of the most recent birth. Since 97.4 % of women were married at the time of birth, we refer to occupation as that of the woman’s husband [34]. Occupations were assigned one of 116 codes according to the classification system of the Israel Central Bureau of Statistics. Occupations were ranked according to the incidence of hospital admission for infant gastroenteritis in 1966–1968, then aggregated into six groups that mirrored those of the British Registrar General’s classification from professionals in group 1 to unskilled manual workers in group 6 [29]. This scale has been used extensively in studying cohort offspring and parents [34], including predicting long-term mortality in women [38]. For this analysis, the six groups were collapsed to three, representing high, middle and low SEP, to allow for comparisons with woman’s education, which was similarly categorized into three groups (0–8 years, 9–12 years and more than 12 years). Examples of occupations in each SEP group include professionals, teachers, and university and yeshiva students classified as high SEP; soldiers, clerks, and skilled agricultural workers classified as middle SEP; and unskilled agricultural and industrial workers classified as low SEP [33].

Additional variables included age at first birth in the cohort (categorized into 5-year intervals, and used as a proxy for the woman’s age at first birth, which may have been prior to 1964), woman’s birth year (dichotomized as before or after 1945, to control for a potential cohort effect) and parity at the last observed birth (categorized as 1, 2–3, and 4 or more children). Ethnic origin was defined as the woman’s country of birth, or her father’s country of birth for women born in Israel, and categorized into four groups (Israel, West Asia, North Africa, Europe etc.). Age at breast cancer diagnosis (categorized into 5-year intervals) and time period of diagnosis (before or after 1995) were abstracted from the cancer registry and included in the survival analysis. Time period of diagnosis was incorporated to account for improved treatment practices and early detection due to mammography.

### Statistical analyses

Two sets of analyses were conducted using similar methods. The first examined the association between SEP and breast cancer incidence for the entire study population. The second examined the association between SEP and all-cause mortality among women diagnosed with breast cancer.

Frequency distributions of independent variables were compared with both measures of SEP and breast cancer outcomes. Person-time incidence and mortality rates were calculated by each SEP measure and incidence density ratios were calculated to compare risk between high and low socioeconomic groups; confidence intervals were computed using the mid-P method. Kaplan-Meier survival curves were plotted and log rank tests used to compare cancer-free survival time and survival time after diagnosis by SEP.

Cox proportional hazard models were constructed to compare the risk of breast cancer and the risk of mortality after diagnosis by levels of SEP and controlling for covariates. The assumption of proportional hazards was confirmed via log-minus-log survival plots for education and occupation. Women with missing parity data, women who developed breast cancer before their first birth in the cohort, and one woman with unknown follow-up time were excluded from this analysis (*n =* 43). Maternal education was imputed as 0–8 years for women with missing data (9.4 %) based on similar baseline characteristics of these two groups; analyses excluding women with missing educational information yielded similar results. Modeling was conducted in stages by adding covariates previously identified as potential confounders. Time period of diagnosis was included in the multivariable survival model due to clinical significance, though it was not significantly associated with SEP. Multiplicative interaction terms were added into each multivariable model to assess effect modification by ethnic origin.

Two sensitivity analyses were conducted for breast cancer incidence. In one, follow-up began 5 years after cohort entry to account for the transient increase in breast cancer risk after pregnancy [39, 40] and to limit the potential of reverse causality. The second was stratified by time period of diagnosis to account for the introduction of the Israeli national mammography program in 1994. We also conducted an additional analysis for both the breast cancer incidence and survival models with mutual adjustment for occupation and education.

Person-time rates and rate ratios were calculated using WinPepi, version 11.39 (J.H. Abramson, 2013). Other analyses used SPSS version 21.0. All tests were two-sided, and a p-value of less than 0.05 was considered to be statistically significant. For the interaction analyses, a p-value of less than 0.01 was considered to be statistically significant.

This study protocol was carried out in compliance with the Helsinki Declaration and approved by the Institutional Review Board at Hadassah-Hebrew University Hospital of Jerusalem and at New York University Langone Medical Center, New York. Permission to use the data was granted by the Hebrew University of Jerusalem.

## Results

### Breast cancer incidence

During 1,493,075 person-years of follow-up (median 37.3 p-y), 2,073 of the 40,586 women (5.1 %) developed breast cancer. The crude incidence rate of breast cancer was 1.39 per 1,000 person-years (95 % CI 1.33 – 1.45). Compared to women who did not develop breast cancer, women with breast cancer were more likely to be older at first birth, have fewer than 4 children, and be of European origin (Table 1). Women of high SEP according to husband’s occupation were 1.3 times more likely to develop breast cancer than women of low SEP (95 % CI 1.17 – 1.48). Women with more than 12 years of education were 1.48 times more likely to develop breast cancer than women with 0–8 years (95 % CI 1.25 – 1.57). There was a moderate correlation between education and occupation, as assessed by Pearson’s correlation coefficient of 0.47 (*p <* .01) (data not shown).

**Table 1:** Characteristics of women of the Jerusalem Perinatal Study cohort (1964–1976) by breast cancer status

Characteristic	Total		Breast cancer diagnosis*			
	(*N* = 40,586)		Yes (*n* = 2073)		No (*n* = 38,513)	
	N	%	N	%	N	%
Age at 1st birth^a^						
<20	3890	9.6	104	5.0	3786	9.8
20-25	16,472	40.6	649	31.3	15,823	41.1
25-30	10,685	26.3	661	31.9	10,024	26.0
30-35	5669	14.0	402	19.4	5,267	13.7
35-40	3030	7.5	208	10.0	2822	7.3
≥40	840	2.1	49	2.4	791	2.1
Ethnic origin						
Israel	5372	13.2	297	14.3	5075	13.2
West Asia	11,484	28.3	584	28.2	10,900	28.3
North Africa	8665	21.3	334	16.1	8331	21.6
Europe	15,065	37.1	858	41.4	14,207	36.9
Birth year						
Before 1945	20,538	50.6	1331	64.2	19,207	49.9
1945 or later	20,048	49.4	742	35.8	19,306	50.1
Parity						
1	8794	21.7	370	17.8	8424	21.9
2-3	18,381	45.3	1,062	51.2	17,319	45.0
≥4	13,374	33.0	640	30.9	12,734	33.1
Unknown	37	0.001	1	0	36	0.1
SEP based on woman’s education						
0-8 years	10,618	26.2	489	23.6	10,129	26.3
9-12 years	14,020	34.5	702	33.9	13,318	34.6
>12 years	12,144	29.9	728	35.1	11,416	29.6
Unknown	3804	9.4	154	7.4	3650	9.5
SEP based on husband’s occupation						
Low	9853	24.3	428	20.6	9425	24.5
Middle	15,456	38.1	806	38.9	14,650	38.0
High	15,277	37.6	839	40.5	14,438	37.5

^**a**^ Range includes right boundary

* All characteristics were significantly associated with breast cancer status (*p <* .001)

**Fig. 1:**
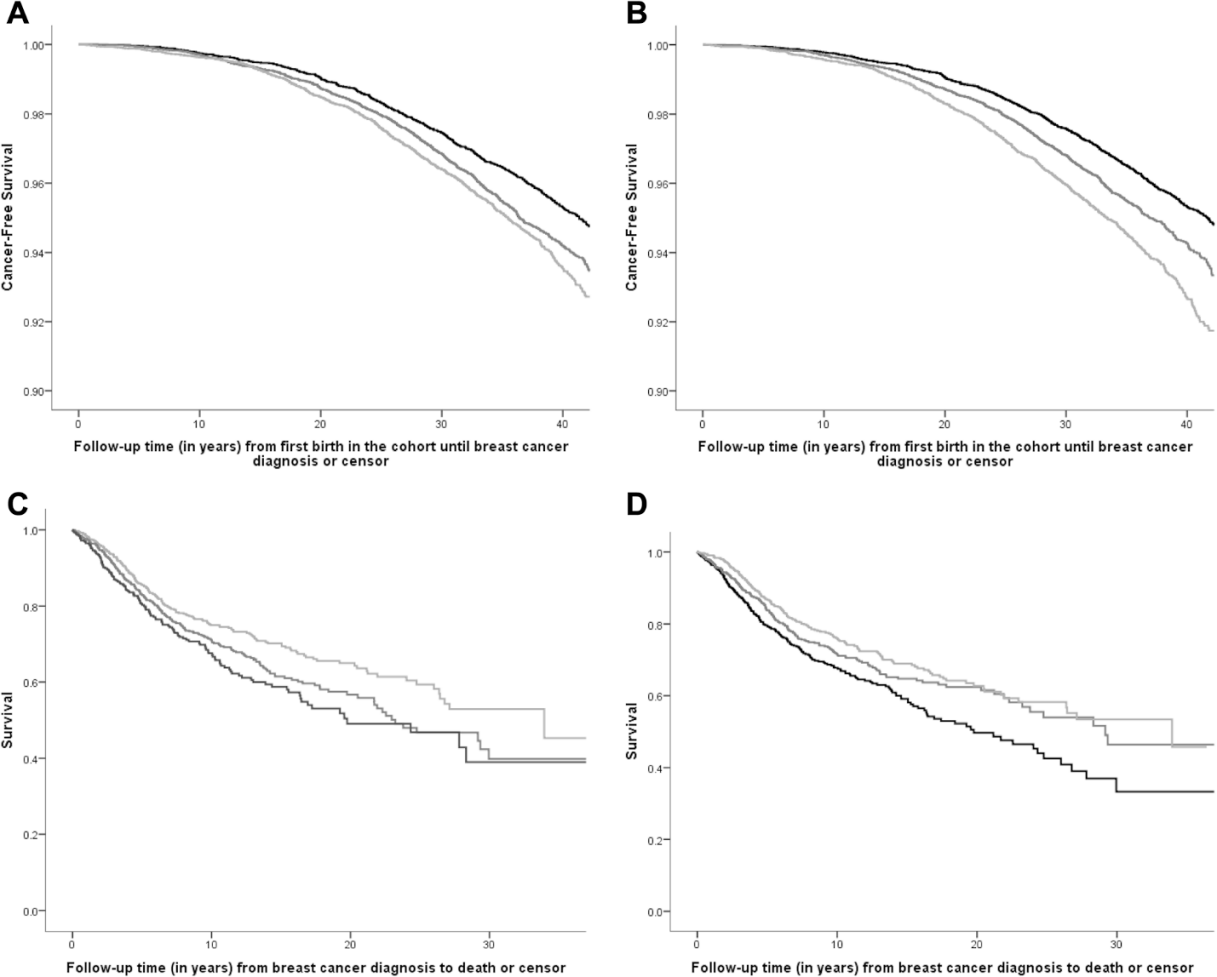
**a-d.**Top Row: Kaplan Meier cancer-free survival curves by **a**) husband’s occupation and **b**) education (*n =* 40,580). Bottom Row: Kaplan Meier survival curves by **c**) husband’s occupation and **d**) education (*n =* 2068). SEP by husband’s occupation: Black = Low; Dark Gray = Middle; Light Gray = High. SEP by education: Black = 0–8 years; Dark Gray = 9–12 years; Light Gray = >12 years

We detected a reversed social gradient in breast cancer incidence by both indicators, with the highest rates of breast cancer in women of high SEP (*p <* .0001) (Fig. 1a-b). Women of low SEP lived longer without being diagnosed with breast cancer than women of high SEP.

Reversed social gradients in breast cancer incidence were retained in models adjusted for reproductive and demographic factors (Table 2). Women of high SEP by occupation had a greater risk of breast cancer (HR 1.42; 95 % CI 1.26 – 1.60) than women of low SEP in the age-adjusted model. After adding parity and ethnic origin, associations were attenuated but remained statistically significant (HR 1.18; 95 % CI 1.03 – 1.35) (Model 2).

**Table 2:** Multivariable-adjusted hazard ratios and 95 % confidence intervals of the association between socioeconomic position and breast cancer risk among the Jerusalem Perinatal Study cohort (1964–1976)

	Age-adjusted^b^		Model 1^c^		Model 2^d^	
	HR	95 % CI	HR	95 % CI	HR	95 % CI
SEP based on husband’s occupation						
Low	1^a^	-	1^a^	-	1^a^	-
Middle	1.29	1.15 – 1.45	1.17	1.03 – 1.31	1.12	0.99 – 1.27
High	1.42	1.26 – 1.60	1.25	1.11 – 1.42	1.18	1.03 – 1.35
SEP based on woman’s education						
0–8 years	1^a^	-	1^a^	-	1^a^	-
9–12 years	1.46	1.31 – 1.63	1.29	1.14 – 1.45	1.27	1.13 – 1.44
>12 years	1.65	1.47 – 1.84	1.41	1.25 – 1.59	1.39	1.21 – 1.60

40,536 women included in each model

^a^ Reference category

^b^ Adjusted for age at 1^st^ birth in the cohort

^c^ Adjusted for age at 1^st^ birth and parity

^d^ Adjusted for age at 1^st^ birth, parity, ethnic origin and time period of birth

Greater inequalities were seen in breast cancer incidence by education. The addition of parity and ethnic origin to the age-adjusted model reduced the magnitude of the association, but a statistically significant increased risk remained among women with 9–12 years (HR 1.27; 95 % CI 1.13 – 1.44) and more than 12 years of education (HR 1.39; 95 % CI 1.21 – 1.60) compared to less educated women (Model 2). We did not detect significant interactions by ethnic origin in models assessing breast cancer incidence by occupation or education.

Results were similar when incidence analyses were conducted with follow-up time starting 5 years after cohort entry. An analysis of breast cancer incidence by time period of diagnosis yielded similar results in the education model among women diagnosed before and after 1994. The occupation model yielded slightly larger gradients in women diagnosed in 1994 or later (HR 1.26; 95 % CI 1.06 – 1.49) compared to before (HR 1.08; 95 % CI 0.87 – 1.32). In the model mutually adjusted for occupation and education, the results for education were similar to the model without adjustment for occupation (HR 1.36; 95%CI 1.18 – 1.57 for women with more than 12 years of education), but the occupational gradient in incidence diminished in magnitude and lost statistical significance (HR 1.09; 95%CI 0.95 – 1.25 for women of high SEP).

### Breast cancer survival

During 19,782 person-years of follow-up from diagnosis (median 7.5 p-y), 598 (28.8 %) of the 2,073 women who developed breast cancer in the cohort died. Women who died were significantly more likely to have completed fewer years of education or be of low SEP by occupation than women who survived (Table 3). Women who died were more likely to be older at first birth, have more children, have been diagnosed at a younger age and before 1995, and less likely to be of European origin than women who survived.

**Table 3:** Characteristics of women of the Jerusalem Perinatal Study cohort (1964–1976) diagnosed with breast cancer by vital status

Characteristic	Total		Vital status*			
	(*N* = 2073)		Deceased (*n* = 598)		Living (*n* = 1475)	
	N	%	N	%	N	%
Age at 1st birth^a^						
<20	104	5.0	30	5.0	74	5.0
20–25	649	31.3	151	25.3	498	33.8
25–30	661	31.9	162	27.1	499	33.8
30–35	402	19.4	150	25.1	252	17.1
35–40	208	10.0	84	14.0	124	8.4
≥40	49	2.4	21	3.5	28	1.9
Age at diagnosis^a^						
<40	181	8.7	112	18.7	69	4.7
40–45	215	10.4	99	16.6	116	7.9
45–50	317	15.3	103	17.2	214	14.5
50–55	421	20.3	103	17.2	318	21.6
55–60	367	17.7	79	13.2	288	19.5
60–65	289	13.9	56	9.4	233	15.8
65–70	171	8.2	25	4.2	146	9.9
≥70	112	5.4	21	3.5	91	6.2
Year of diagnosis						
Prior to 1995	907	43.8	437	73.1	470	31.9
1995 or later	1166	56.2	161	26.9	1005	68.1
Ethnic origin						
Israel	297	14.3	102	17.1	195	13.2
West Asia	584	28.2	169	28.3	415	28.1
North Africa	334	16.1	105	17.6	229	15.5
Europe	858	41.4	222	37.1	636	43.1
Birth year						
Before 1945	1331	64.2	430	71.9	901	61.1
1945 or later	742	35.8	168	28.1	574	38.9
Parity						
1	370	17.8	84	14.0	286	19.4
2–3	1062	51.2	289	48.3	773	52.4
≥4	640	30.9	224	37.5	416	28.2
Unknown	1	0.1	1	0.2	0	0.0
SEP based on woman’s education						
0–8 years	489	23.6	160	26.8	329	22.3
9–12 years	702	33.9	196	32.8	506	34.3
>12 years	728	35.1	182	30.4	546	37.0
Unknown	154	7.4	60	10.0	94	6.4
SEP based on husband’s occupation						
Low	428	20.6	142	23.7	286	19.4
Middle	806	38.9	247	41.3	559	37.9
High	839	40.5	209	34.9	630	42.7

^**a**^ Range includes right boundary

* All characteristics were significantly associated with vital status (*p <* .05)

The overall mortality rate among women diagnosed with breast cancer was 30.18 per 1,000 person-years (95 % CI 27.83 – 32.67). Women of low SEP by occupation were 1.49 times more likely to die after a breast cancer diagnosis than women of high SEP (95 % CI 1.12 – 1.84). Women with 0–8 years of education were 1.54 times more likely to die after being diagnosed with breast cancer than women with more than 12 years (95 % CI 1.25 – 1.86) (data not shown).

Women of high SEP had greater survival time after diagnosis than women of low SEP by both indicators (*p <* .01), as seen in Fig. 1c-d.

Women of low SEP had a greater risk of mortality after a breast cancer diagnosis than women of high SEP in the models adjusted for demographic, reproductive and diagnostic factors (Table 4). Women of low SEP by occupation had a statistically significant increased risk of death (HR 1.33; 95 % CI 1.04 – 1.70), and women of middle SEP had a greater risk compared to women of high SEP that bordered statistical significance (HR 1.22; 95 % CI 1.00 – 1.48).

**Table 4:** Multivariable-adjusted hazard ratios and 95 % confidence intervals of the association between socioeconomic position and breast cancer survival, Jerusalem Perinatal Study cohort (1964–1976)

	Age-Adjusted^b^		Model 1^c^		Model 2^d^	
	HR	95 % CI	HR	95 % CI	HR	95 % CI
SEP based on husband’s occupation						
Low	1.50	1.21 – 2.86	1.34	1.07 – 1.68	1.33	1.04 – 1.70
Middle	1.27	1.06 – 1.53	1.24	1.03 – 1.49	1.22	1.00 – 1.48
High	1^a^	–	1^a^	–	1^a^	–
SEP based on woman’s education						
0–8 years	1.59	1.30 – 1.93	1.40	1.13 – 1.74	1.39	1.07 – 1.79
9–12 years	1.16	0.94 – 1.42	1.18	0.96 – 1.44	1.16	0.93 – 1.44
>12 years	1^a^	–	1^a^	–	1^a^	–

2068 women included in each model

^a^ Reference category

^b^ Adjusted for age at diagnosis

^c^ Adjusted for age at diagnosis, age at first birth and parity

^d^ Adjusted for age at diagnosis, age at first birth, parity, ethnic origin and time period of diagnosis

Women with 0–8 years, but not women with 9–12 years, had a greater risk of mortality compared to women with more than 12 years of education. In the fully-adjusted model (Model 2), women with 0–8 years had an increased risk of death of almost 40 % (HR 1.39; 95 % CI 1.07 – 1.79) compared to women with more than 12 years of education.

In an analysis mutually adjusted for education and occupation, the increased risk of mortality among women of low SEP slightly reduced in magnitude for both occupation and education and were no longer statistically significant (data not shown).

**Fig. 2:**
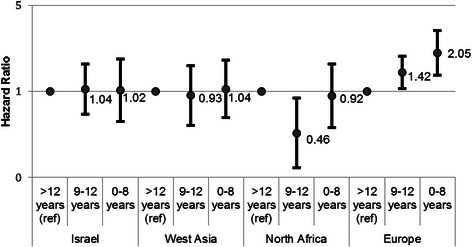
Hazard ratios and 95 % confidence intervals of death from breast cancer by woman’s educational attainment, stratified by ethnic origin, in the Jerusalem Perinatal Study cohort (1964–1976). Reference category is women with more than 12 years of education

We detected a multiplicative interaction between education and ethnic origin in breast cancer survival (*p for interaction =* .005) in the fully-adjusted model (Fig. 2), but interaction by origin was not seen among occupational groups. Women of European origin with 0–8 years of education had a two-fold greater risk of mortality (HR 2.05; 95 % CI 1.35 – 3.12), and those with 9–12 years had a 42 % increased risk of mortality (HR 1.42; 95 % CI 1.05 - 1.93) compared to women with more than 12 years. Social gradients were not observed within the other ethnic groups, though a statistically significant decreased risk was observed among women of North African origin with 9–12 years of education compared to more than 12 years (HR 0.46; 95 % CI 0.24 – 0.88).

## Discussion

In our population-based cohort, women of high SEP, as measured by the woman’s education and her husband’s occupation, had a greater risk of developing breast cancer than women of low SEP, consistent with previous findings [1, 3, 10, 41, 42] but novel for the study population of Israeli Jewish women. A social gradient was detected in breast cancer survival with less affluent women by husband’s occupation at a greater risk of all-cause mortality after a breast cancer diagnosis than more affluent women, which supports previous work [15, 17]. Education exhibited a threshold association with breast cancer survival. Only women with low education (0–8 years) had a greater risk of mortality than women with high education (more than 12 years), as opposed to the gradient effect observed by others [2, 3, 15, 17, 42]. The association between education and breast cancer survival was modified by ethnic origin. An educational gradient was observed among women of European origin, while women of Asian, North African and Israeli origin showed no trend.

The reversed social gradient in breast cancer risk may be explained by other variables not available in this analysis, such as BMI and exogenous hormone use, which have partially explained the association between SEP and incidence in previous studies [9, 41]. In Israel, higher levels of education are associated with higher rates of OC use [43], late age at last birth [44] and lower likelihood of breastfeeding [45], which may explain the greater risk of breast cancer among women of high SEP. The increased risk of breast cancer mortality detected among women of low SEP in this analysis was in line with previous studies that included similar covariates [2, 3]. Studies that included additional diagnostic and prognostic characteristics, such as stage at diagnosis and estrogen receptor status, found that these variables attenuated the greater risk among women of low SEP [1, 17, 20, 24].

While in Israel, screening, diagnosis, and treatment services are available to citizens free of charge under the National Health Law, there are probably inequalities in healthcare utilization that affect breast cancer incidence. Women of low SEP in Israel were less likely to undergo regular mammography than women of high SEP [46], though recent national breast cancer trends suggest that early detection programs have reached more women from lower socioeconomic levels [47]. A sensitivity analysis indicated slightly larger gradients in incidence in women diagnosed after the onset of the national mammography program for occupation only. Once diagnosed, it is less likely that survival differences are due to disparities in treatment.

We found that education was associated with larger inequalities in breast cancer incidence and survival than occupation. Analyses mutually adjusted for occupation and education also suggested that education has a stronger association with breast cancer outcomes than occupation. A population-based Danish study also determined that education had the strongest association with incidence, but found inequalities of similar magnitude in survival across socioeconomic indicators [3]. Differences in the associations between SEP and breast cancer outcomes by indicator suggest that education and occupation may influence breast cancer through different pathways. Occupation, a prestige-based measure, is a reflection of social standing, while education is a material-based measure reflecting parental SEP, early life and adult resources [27]. In this study, we included the husband’s occupation, reflecting household SEP, rather than the occupation of the woman, as 59 % of women in the cohort did not work outside the home at the time of childbirth. Divorce rates were low in Israel during this time period, indicating stable partnerships [48]. The stronger effect of education as compared to occupation may be because occupation did not capture the SEP of the woman as well as the individual-level variable of education. The original six-group scale of occupation was also collapsed into three groups for the analysis in order to meet the proportional hazards assumption; heterogeneity within the three occupational groups could contribute towards the smaller magnitude of association observed with the occupation indicator.

The knowledge and skills acquired through education may directly affect health behavior, especially in conditions where more is known about treatment and prevention such as breast cancer [48]. The stronger effect of education suggests that differences in health behaviors may explain the observed disparities in incidence and survival in this study. If so, this finding has important policy implications, suggesting that health promotion programs should be tailored based on education level. Highly educated women may benefit more from programs that emphasize risk reduction, such as the promotion of breastfeeding and physical activity, while women with less education would benefit more from campaigns to increase mammography utilization.

The literature on the interaction between race/ethnicity, SEP and breast cancer survival is not extensive and results have been inconsistent [22, 23, 49, 50]. In the United States, a recent study found a significant interaction between race/ethnicity and SEP on mortality among women with stage 2–3 breast cancer, with black women at a greater risk of mortality than white women among the intermediate and most affluent groups, but not the least affluent group [23]. Others have not detected significant interactions in breast cancer survival [22, 49, 50].

The interaction between ethnic origin, SEP and breast cancer survival has not been previously examined among Israeli Jews. The educational gradient detected within women of European origin could be due to chance, but this seems unlikely given the sample size and strength of the association (*n =* 858). The decreased risk observed among women of North African origin with 9–12 years of education compared to more than 12 years was likely a chance finding due to small sample size (*n =* 334). The interaction could be explained by an unmeasured confounder like religiosity, which has been associated with lower educational attainment and lower rates of mammography among Israeli Jews [51], and is associated with European origin in the cohort [34]. Jewish women of European origin also differ from Jewish women of other ethnicities due to a higher prevalence of BRCA mutations [52] that may affect survival [53]; to our knowledge, the association between SEP and breast cancer survival has not been investigated within carriers. Given this novel finding, more research may be warranted to examine risk factors that may explain the sizable social gradient observed among the high-risk group of European women.

This study is limited by data availability, which was restricted to information collected on the entire cohort from birth notifications. Additional information that may be related to breast cancer risk, such as family history and BMI, was not available. Parity and age at first birth were censored, which may have underestimated their effects due to non-differential misclassification. Generalizability of the results may be limited to parous women only since nulliparous women were not included in the cohort; however, only 5.6 % of married Israeli Jewish women did not have children during this time period [54]. In the survival analysis, all-cause mortality after a breast cancer diagnosis was used instead of cause-specific mortality, which was not available for all women. The use of all-cause mortality could lead to an overestimate of the association with survival, particularly for diseases that generally have good prognoses. However, all-cause mortality can be preferable over cause-specific mortality due to potential errors in cause-of-death reporting on death certificates [55, 56]. Finally, the survival analysis was limited by a lack of information on prognostic factors, such as stage at diagnosis, which have partially explained social gradients in breast cancer survival in previous studies [1, 17, 20, 24].

The main strength of this analysis is the use of high-quality data, abstracted from government and medical records, for a population-based cohort. Other strengths include the large sample size, lengthy follow-up with minimal loss, and high case ascertainment due to linkage with population registries. Finally, we were able to examine breast cancer incidence and survival within the same cohort of women and make comparisons between SEP measures with these two outcomes due to the availability of two individual-level socioeconomic indicators.

## Conclusions

We detected a positive association between SEP and breast cancer incidence and survival by two socioeconomic indicators. Associations were stronger when education, as opposed to occupation, was used to measure SEP. This contributes to the literature on the comparative effect of socioeconomic indicators on breast cancer outcomes and suggests that a behavioral mechanism may explain breast cancer disparities. We identified high-risk socioeconomic groups that need attention to reduce the burden of breast cancer incidence and improve survival. We enrich the literature in disentangling the associations between SEP, race/ethnicity and breast cancer survival, and suggest a future area of research to explain the robust educational gradient we detected among the high-risk group of European women.

## Abbreviations

BMI: Body mass index
CI: Confidence interval
OC: Oral contraceptive
HR: Hazard ratio
SEP: Socioeconomic position
